# Engineering *Aspergillus oryzae* A-4 through the Chromosomal Insertion of Foreign Cellulase Expression Cassette to Improve Conversion of Cellulosic Biomass into Lipids

**DOI:** 10.1371/journal.pone.0108442

**Published:** 2014-09-24

**Authors:** Hui Lin, Qun Wang, Qi Shen, Junwei Ma, Jianrong Fu, Yuhua Zhao

**Affiliations:** 1 Institute of Environment Resource and Soil Fertilizer, Zhejiang Academy of Agriculture Science, Hangzhou, China; 2 Institute of Microbiology, College of Life Sciences, Zhejiang University, Hangzhou, China; University of Wisconsin - Madison, United States of America

## Abstract

A genetic modification scheme was designed for *Aspergillus oryzae* A-4, a natural cellulosic lipids producer, to enhance its lipid production from biomass by putting the spotlight on improving cellulase secretion. Four cellulase genes were separately expressed in A-4 under the control of *hlyA* promoter, with the help of the successful development of a chromosomal genetic manipulation system. Comparison of cellulase activities of PCR-positive transformants showed that these transformants integrated with *celA* gene and with *celC* gene had significantly (*p*<0.05) higher average FPAase activities than those strains integrated with *celB* gene and with *celD* gene. Through the assessment of cellulosic lipids accumulating abilities, *celA* transformant A2-2 and *celC* transformant D1-B1 were isolated as promising candidates, which could yield 101%–133% and 35.22%–59.57% higher amount of lipids than the reference strain A-4 (WT) under submerged (SmF) conditions and solid-state (SSF) conditions, respectively. Variability in metabolism associated to the introduction of cellulase gene in A2-2 and D1-B1 was subsequently investigated. It was noted that cellulase expression repressed biomass formation but enhanced lipid accumulation; whereas the inhibitory effect on cell growth would be shielded during cellulosic lipids production owing to the essential role of cellulase in substrate utilization. Different metabolic profiles also existed between A2-2 and D1-B1, which could be attributed to not only different transgene but also biological impacts of different integration. Overall, both simultaneous saccharification and lipid accumulation were enhanced in A2-2 and D1-B1, resulting in efficient conversion of cellulose into lipids. A regulation of cellulase secretion in natural cellulosic lipids producers could be a possible strategy to enhance its lipid production from lignocellulosic biomass.

## Introduction

Biodiesel is widely recognized as a type of green fuel, which has advantages of low sulfur content, being non-toxic and biodegradable, lack of aromatics, and excellent lubricity [1]. Today, the shortage of less-cost oils, which can be used as a raw material in biodiesel production, has become a major obstacle in the development of biodiesel market. It is essential and urgent to explore sustainable and low-priced oil feedstocks for the wide use of biodiesel.

Single cells oils (SCOs) are triacylglycerols from renewable biomass, which have been well documented to be a promising feedstock for biodiesel production. SCOs can be produced by some oleaginous microorganisms, such as yeast, fungi, bacteria and microalgae, through microbial fermentation. A lot of efforts have been done all over the world to reduce the production cost during SCOs production and to explore cheap and abundant substrates for oleaginous microorganism cultivation. The social and economic benefits of producing SCOs from lignocellulosic biomass instead of crops are widely appreciated. Testing strategies to establish more efficient, less-cost and sustainable technologies based on microbial fermentation for SCOs production from lignocellulosic biomass is under current investigation by various start-up biotechnology companies and research centers [2], [3], [4], [5], [6]. Among these researches and technologies, direct microbial conversion of lignocellulosic biomass into lipids, as an example of consolidated bioprocessing (CBP), is considered to be economically attractive for “third generation” biofuel production due to its simple feedstock processing and low energy inputs [7]. To do so, hydrolysis of cellulose and hemicelluloses in biomass and production of valuable product, which were currently accomplished in different reactors or different organisms, are combined in a single process step in CBP. During the lipid production process in CBP, the substantial capital and material expense occurred in the enzymes preparation could be avoided.

Integration of cellulose hydrolysis and lipid biosynthesis by mixing cellulolytic enzymes with auxiliary nutrients in a single bioreactor is an example of CBP for lipid production from lignocellulosic materials [4], [8]. However, the high cost of the cellulolytic enzymes is the primary hindrance in this cellulosic lipids production. Isolation or engineering of microorganisms, which could de-polymerize biomass polysaccharides to fermentable sugars efficiently and convert this mixed-sugar hydrolysate into cellular lipids effectively, is recognized to be another example of CBP to produce lipids directly from plant biomass. In recent years, microbial species capable of converting cellulose into microbial lipid directly, such as *Aspergillus oryzae* A-4 [9], *Microsphaeropsis* sp. [6], *Colletotrichum* sp. and *Alternaria* sp. [10] have been successfully isolated from natural environments. Several recombinant oleaginous CBP producers are also being reported after metabolic engineering to incorporate the feature of naturally biomass polysaccharides utilization into oleaginous cell factories [3], [11], [12]. In the report of Hetzler et al. [12], introduction of a cellobiose utilization pathway enabled *Rhodococcus opacus* PD630 to accumulate fatty acids up to 39.5±5.7% (wt/wt) of cell dry mass from cellobiose substrates. One-step production of lipids from birch cellulose was demonstrated to be feasible by co-fermentation of the recombinant cellulose degrading strain of *R. opacus* PD630 and the recombinant cellubiose-utilizing strain of *R. opacus* PD630 [3]. Nevertheless, the reports regarding construction of oleaginous CBP producers are still limited. It is indispensible to carry out more studies for the purpose of exploring new strains, improving currently available strains and optimizing the fermentation conditions.

In our previous work [9], a cellulolytic and oleaginous strain of *Aspergillus oryzae* A-4 was isolated from soil environments. The biochemical approaches by controlling the nutritional and cultivation conditions to channel metabolic flux into lipid biosynthesis has been performed to optimize the lipid production of *A. oryzae* A-4 under solid-state conditions with wheat straw and bran mixture as substrates. Although these attempts are promising, the current lipid conversion efficiency of *A. oryzae* A-4 is still far from the requirements from commercially viable production. Considering various aspects of a SCOs CBP scheme, here we sought to modify the cellulase secretion of strain A-4 for the exploration of recombinant CBP producers with enhanced lipid production from lignocelluloses. It is known that the biochemical event associated with the lipid production in direct microbial conversion system with cellulosic wastes as substrates is composed of three steps: polymers decomposition, monomer uptake, and lipid biosynthesis. In such cases, the hydrolytic activity of the microbes, which is related to the polymers decomposition, could associate closely with the storage lipid accumulation [9], [13]. Thus, it could be speculated that insufficient available sugars for *A. oryzae* A-4 to take up is a major obstacle for its high lipid yielding. Heterogeneous cellulase expression in this study was controlled under the promoter from a hemolysin-like protein gene (*hlyA* gene promoter). It has been demonstrated that high-level protein expression under the control of *hlyA* promoter can not be repressed by glucose not only in solid-state culture of *A. oryzae* but also in liquid culture [14]. *A. oryzae* A-4 is a good platform that can be engineered for an efficient CBP scheme after only several modifications rather than de novo introduction of complete metabolism pathway. Actually, it is difficult to engineer a microorganism with all of the desired features necessary for an efficient CBP scheme so far [15].

## Materials and Methods

### Strains

The oleaginous fungus *A. oryzae* A-4, which was previously isolated by Lab of Microbiology, Zhejiang University [9], was used as a recipient for transformation and chromosomal DNA preparation in this work. *A. oryzae* A-4 were maintained on Czapek-Dox (CD) plate (0.6% NaNO_3_, 0.052% KCl, 0.152% KH_2_PO_4_, 0.052% MgSO_4_·7H_2_O, 1% glucose and pH 6.5) at 4°C before use. The *Escherichia coli* DH5α used for plasmid recovery and cloning experiments was grown in Luria-Bertani broth (LB).

### Plasmids construction

The genes and plasmids used in this study are summarized in Table 1. Primers were listed in Table S1. The construction procedure of the recombinant expression vector for *A. oryzae* A-4 was described below. The DNA fragment of *amyA* terminator was first generated by polymerase chain reaction (PCR) from the genomic DNA of *A. oryzae* ATCC 42149. The vector pPTRI-Tamy was then constructed by the ligation of the *amyA* terminator fragment with the parent vector pPTR I (*Takara* Bio Inc.). Construction of plasmids expressing target genes under the control of the *hlyA* promoter was performed by using overlap PCR, which comprised of two PCR steps. In the first PCR step, the target gene fragment and its corresponding *hlyA* promoter fragment was generated by PCR, respectively. For each molecule, the primer at the end to be joined is constructed such that it has a 5′ overhang complementary to the end of the other molecule. In the second step, the target gene fragment and its corresponding *hlyA* promoter fragment are mixed, and a PCR was carried out with only the primers for the far ends. The overlapping complementary sequences introduced will serve as primers and the two sequences will be fused. To test if the expression vector could be used for foreign protein expression, eGFP expression vector was first constructed for transformation. To obtain the eGFP expression vector, the primer set of FP2-GFP-overlap/RP2-GFP-Rec and FP1-hlyA-Rec/RP1-hGFP-overlap was used for the isolation of the *eGFP* fragment (AGX13949) from vector PET-eGFP (previously constructed in our lab) and its corresponding *hlyA* promoter fragment from *A. oryzae* ATCC 42149 genomic DNA, separately. Similarly, the primer sets of FP2-CelA-overlap/RP2-CelA-Rec + FP1-hlyA-Rec/RP1-hCelA-overlap; FP1-hlyA-Rec/RP1-hCelB-overlap + FP2-CelB-overlap/RP2-CelB-Rec; FP1-hlyA-Rec/RP1-hCelD-overlap + FP2-CelD-overlap/RP2-CelD-Rec and FP1-hlyA-Rec/RP1-hCelC-overlap + FP2-CelC-overlap/RP2-CelC-Rec were designed for the construction of CelA, CelB, CelD and CelC expression vectors, respectively. All DNA fragments for the construction of CelA, CelB, CelD and CelC expression vectors were cloned from *A. oryzae* ATCC 42149 genomic DNA. The ligation of the vector-specific fragment with the vector pPTRI-Tamy was achieved by using CloneEZ^®^ PCR Cloning Kit (GenScript). In the final construct, the separate target gene (i.e. *celA*, *celB*, *celD*, *celC* or *eGFP*) was cloned downstream of the *hlyA* promoter and upstream of the *amyA* terminator in vector pPTR I. All PCRs were carried out with PrimeSTAR HS DNA Polymerase (Takara Bio Inc.). Nucleotide sequences of constructed plasmids were sequenced in Shanghai Sangong Co., Ltd.

**Table 1:** Genes and plasmids used in this study.

Gene/plasmid	Function/comments	Source/reference
Gene		
*celA* (AO090026000102)^a^	Cellulase gene	[14], [24]
*celB* (AO090010000314)^a^	Cellulase gene	[14], [24]
*celD* (AO090012000941)^a^	Cellulase gene	This work
*celC* (AO090001000348)^a^	Cellulase gene	This work
*eGFP*	Enhanced Green Fluorescent Protein gene	[29]
*hlyA* promoter	Promoter region of *hlyA* (a hemolysin-likeprotein gene, AO090010000018^a^)	[14]
*amyA* terminator	Terminator region of *amyA* (a taka-amylasegene, AO090023000944^a^)	[29], [30]
Plasmid		
pPTRI	A chromosomal integrating and*E. coli*-*Aspergillus* shuttle vector	[17]
pPTRI-Tamy	Vector pPTRI containing *amyA* terminator	This work

a DOGAN accession number (DOGAN, http://www.bio.nite.go.jp/dogan/).

### Protoplasts preparation, transformation and PCR checking

The pyrithiamine-resistant transformation system was applied for the strain A-4, and each expression vector was chromosomally integrated into A-4 according to Protoplast-PEG method as previously described but with slight modification [16], [17]. Spores of *A. oryzae* A-4 were inoculated on PDA plates at 30°C for 3–4 days. 1 ml of spores suspension with a spore concentration of 1×10^7^ ml^−1^ was then collected from the plates and inoculated into 100 ml of CD liquid medium. Incubation was performed in an orbital shaker at 180 rpm and 30°C for 20–24 hours. The germinated spores were washed with sterilized distilled water and then resuspended in 5 ml of protoplast forming solution (0.8 M NaCl, 10 mM Na phosphate buffer, pH 6.0) containing Yatalase (Takara Bio Inc.) at a concentration of 20 mg ml^−1^. The suspension was incubated at 30°C for 2–2.5 hours to allow the release of the protoplasts. The protoplasts were subsequently collected and washed twice with 0.8 M NaCl. The collected protoplasts were resuspended in S1 (0.8 M NaCl, 10 mM CaCl_2_, 10 mM Tris-HCl, pH 8.0) plus 1/5 of the final volume of S2 (40% (w/v) PEG 4000, 50 mM CaCl_2_, 50 mM Tris-HCl, pH 8.0), at a concentration between 1×10^7^ and 1×10^9^ protoplasts ml^−1^. 0.2 ml of the protoplast suspension were mixed with 10–15 µl (1–10 µg) of plasmid and then maintained on ice for 30 min. After that, 1 ml of S2 was added and the mixture was incubated at room temperature for 15 min. The mixture was diluted with 8.5 ml of S1 and the protoplast was collected by centrifugation before resuspended in 0.2 ml of S1. The protoplast suspension was poured as an overlay on CD selection soft plates containing 0.1 mg l^−1^ pyrithiamine hydrobromide (PT-h, Sigma). The plates were incubated at 30°C for 5–7 days in order to allow the regeneration of the protoplasts. Spores from single colonies were collected as described above and stored at –80°C for further analysis. PCR checking was carried out for initial screening of positive transformants. In most cases, the fusion fragments consisting of *hlyA* promoter and target genes could be obtained from the chromosomal DNA of the positive transformant by using primers in overlap PCR for the far ends (Table S1), while no products could be observed from that of host strain using the same primers.

### Microscopic observations

Conidia of recombinant strains harboring the eGFP expression cassette were inoculated into CD selection plates with 0.1 mg l^−1^ PT-h. Cells were grown at 30°C for 3 days, after that the fungal hyphae was placed onto a slide for observation under fluorescence to measure the fluorescence expression of *eGFP* transformants. Lipid accumulation of the wild-type A-4 can be initiated on a nitrogen-limited solid medium as described in our previous work [9]. After 4–6 days’ cultivation, the fungal mycelia was taken and mixed with Nile red solution (final concentration in acetone: 0.25 µg ml^−1^). Fluorescence observation was performed immediately on the mycelia stained with Nile red. All the observations were performed by using an Eclipse 80i microscope (Nikon) equipped with Plan APO VC 100X/1.40 oil objective.

### Southern blot analysis

The purified genomic DNA was digested with *Hind* III and separated by agarose gel electrophoresis. The genomic DNA fragments were transferred onto a positively charged nylon membrane, Hybond-N+ (Amersham Pharmacia Biotech). A 0.7-kb PCR amplified fragment containing the open reading frame (ORF) of Ampicillin resistance gene (*Amp* ^r^) was used as a probe. Probe labeling and blot detection were performed using the DIG High Prime DNA Labeling and Detection Starter Kit II (Roche, Mannheim, Germany) according to the manufacturer’s instructions.

### Fermentation experiments

Submerged fermentation (SmF) for SCOs production was carried out in basal medium [18] containing (g L^−1^): KH_2_PO_4_, 2.0; (NH_4_)_2_SO_4_, 2.1; MgSO_4_·7H_2_O, 0.3; CaCl_2_·7H_2_O, 0.3; MnSO_4_·H_2_O, 0.00156; ZnSO_4_·7H_2_O, 0.0014; CoCl_2_·6H_2_O, 0.00266; yeast extract, 0.5; pH 5.5; supplemented with 2% of carbon source. Carbon sources used for SmF experiments involved glucose, maltose, avicel and wheat straw. All the SmF experiments were carried out with four replications and at 30°C and 180 rpm for 4 days. The SSF medium consisted of 3.6 g wheat straw, 0.4 g wheat bran, and 4 ml basal medium in Petri dishes (U = 9 cm). After sterilization, the media was cooled down and inoculated with 0.5 ml inoculums per gram of dry mass and static cultivated at 30°C, 50%–80% humidity. All the SSF experiments were carried out with three replications. Spore suspension (1×10^6^ spore ml^−1^) was used as the inoculums in this work. Wheat straw and bran were dried at 80°C for 4 h and milled to 20–40 meshes before use.

#### Ethics statements

Wheat straw, collected from Bozhou city, Anhui province (GPS coordinates: 33.923136, 115.83361) was the agricultural waste, and no specific permissions were required for the sampling activity. The field studies did not involve endangered or protected species.

### Analytical methods

The supernatants of the fermented SmF media were collected for enzyme assay after centrifugation, while the solid residues were gathered and dried for a lipid assay [19] and a biomass assay based on glucosamine estimation of the fungal cell wall [20]. The total lipid in SSF was extracted and determined as described in our previous report [9]. The percentage of oil in relation to the dry matter (w/w) was expressed as lipid content. The fungal biomass in SSF was determined by the same method [20] as described in SmF, and the biomass from SSF experiments was depicted as mg glucosamine per gram dry substrate (mg gds^−1^). For enzyme assay, the crude enzymes should be first extracted from the solid-state fermented products. Extraction was performed at 37°C for 2 hours by mixing 1 gram of fermented residue with 20 ml of citric acid buffer (pH 4.8). The cellulase activity in the crude enzyme extract was measured according to the method of Ghose [21] by determination of filter paper cellulase (FPA) activity and carboxymethylcellulase (CMCase) activity. The release of reducing sugars was assayed using 3, 5-dinitrosalicylic acid (DNS) method. One unit of FPA activity (IU) and CMCase activity (IU) was defined as the amount of enzymes required to release 1 µmol of substrate per minute under assay conditions. The protein concentration in the crude enzyme extract was quantified by the Bradford assay [22]. Loss in dry matter (LDM) in SSF was obtained using the following equation:
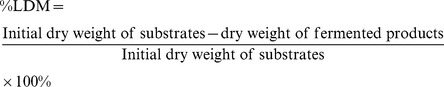



## Results and Discussion

### Development of a system for chromosomal manipulation in *A. oryzae* A-4

**Figure 1:**
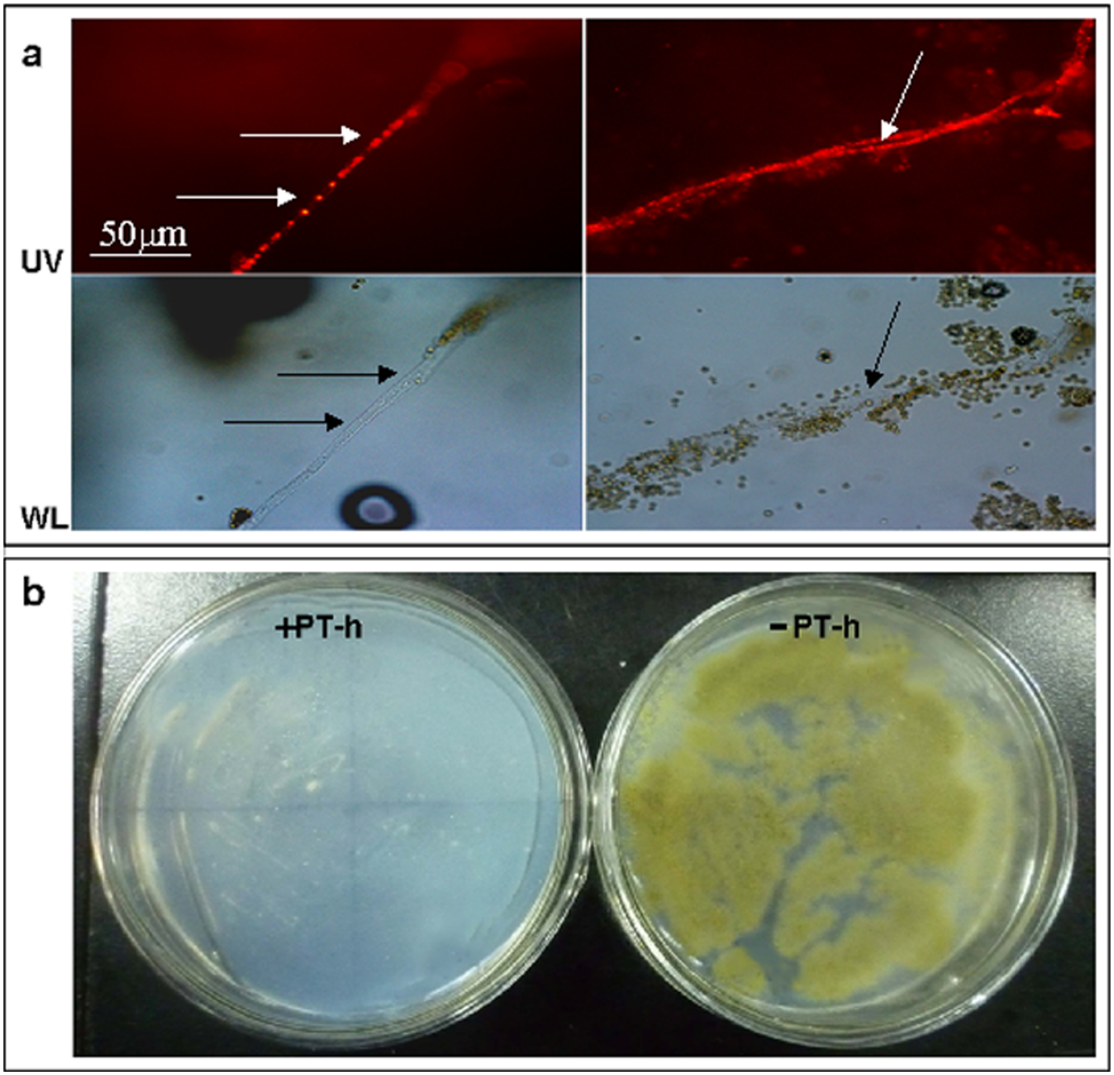
Lipid droplets accumulated in the mycelium of the reference strain A-4 (A) and the cell growth of strain A-4 grown on CD-plate without (-PT-h) and with 0.1 mg l^−1^ PT-h (+PT-h) (B). (A) Strain A-4 was cultivated on wheat straw and bran mixture for 6 days. The Nile red stained fermented products were then microscopic observed under ultraviolet (UV) and white light (WL); (B) Inhibitory effects of PT-h on the cell growth of A-4. Incubation was performed at 30°C for 5 days.

In order to construct a transformation system for genetic manipulation, it is necessary to screen a drug exhibiting effective inhibitory effects on host strains and to obtain its corresponding resistance gene. Compared with bacteria, filamentous fungi have a stronger tolerance to many types of drugs [23], which would result in some difficulties for their genetic manipulation. Pyrithiamine is a potent antagonist of thiamine. In 2002, Japanese researchers found that cell growth of a majority of detected filamentous fungi, including *Aspergillus oryzae* RIB138, *Aspergillus niger* IAM2561, *Aspergillus nidulans* FGSC89 and *Trichoderma reesei* IFO31326, could be suppressed when more than 0.1 mg l^−1^ of pyrithiamine was added into the culture medium [17]. Kubodera et al. [17] then constructed the *E. coli*-*Aspergillus* shuttle vector pPTR I and pPTR II, and the target filamentous fungi were found to be insensitive to pyrithiamine after the transformation of pPTR I or pPTR II. In this work, pyrithiamine hydrobromide (PT-h) was used instead of pyrithiamine as the selection pressure to construct a transformation system for *A. oryzae* A-4, which can accumulate a high quantity of cellular lipids using cellulosic materials as the major substrate (Fig. 1a). As shown in Fig. 1b, the cell growth of wild-type A-4 presented well on the CD solid medium without PT-h but was completely inhibited with 0.1 mg l^−1^ PT-h. The result indicated that PT-h exhibited a similar result to pyrithiamine. 0.1 mg l^–1^ PT-h can be used as selection pressure for *A. oryzae* A-4.

**Figure 2:**
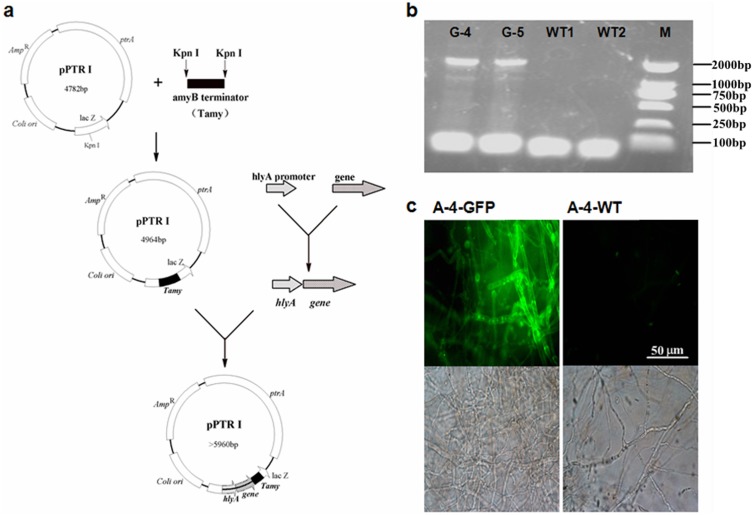
Recombinant strains engineered to express eGFP protein. (A) Construction of the recombinant expression vector for *A. oryzae* A-4; (B) Isolation of positive transformants by PCR checking; (C) Microscopic observations of mycelium samples exposed to ultraviolet (UV) and white light (WL), which collected from the transformant G-4 (A-4-GFP) and the wild-type A-4 (A-4-WT), respectively.

The eGFP expression vector of pPTRI-Tamy-hlyA-eGFP, which comprised of the *hlyA* promoter, the *amyB* terminator, the pyrithiamine resistant gene (*ptrA*) and the target *eGFP* gene, was constructed according to the procedure described in Fig. 2a. Transformants harboring eGFP expression cassette, which exhibited well growth performances on the CD solid media with PT-h, were subsequently isolated and used for PCR checking. No PCR products of the expect size, namely the fusion fragment consisting of *hlyA* promoter and *eGFP* gene (P*hlyA*-*eGFP* fusion gene), can be amplified from the genomic DNA of strains without the integration of the vector pPTRI-Tamy-hlyA-egfp (Fig. 2b). The transformants with PCR products amplified from their genomic DNA were considered as the positive transformants (Fig. 2b). Further investigation of the PCR-positive transformants using fluorescence microscopy (Fig. 2c) showed that an obvious eGFP-fluorescence could be observed in the mycelia of transformant G-4 but hardly found in the mycelia of the reference strain A-4. The result demonstrated that the above-described manipulation procedure can be used for the chromosomal genetic manipulation in *A. oryzae* A-4. Indeed, a proper genetic manipulation system is necessary not only for heterogeneous protein expression in *A. oryzae* A-4 but also for the regulatory mechanisms elucidation during cellulosic lipids accumulation.

### Construction of recombinant strains with increased cellulase activities

According to the procedure described in Fig. 2a, four recombinant vectors separately harboring *celA* gene, *celB* gene, *celD* gene and *celC* gene were constructed and introduced into the host strain *A. oryzae* A-4. For each recombinant vector, more than ten transformants were selected after several generations. The genomic DNAs from all transformants and the wild-type strain A-4 were extracted and used as templates for PCR checking to demonstrate whether the cellulase expression cassette was successfully integrated into the host genome. The size of PCR products (P*hlyA*-*celA/celB/celD/celC* fusion gene) obtained from the genomic DNA of transformants harboring *celA* gene, *celB* gene, *celD* gene and *celC* gene were approximately 1.8 kb, 2.3 kb, 2.6 kb and 2.4 kb, respectively; no PCR products could be obtained from wild-type A-4 (Fig. S1). Overall, five positive *celA* transformants, five positive *celB* transformants, six positive *celD* transformants and six positive *celC* transformants were isolated for further investigations owing to the PCR results.

SmF of wheat straw by these isolated transformants was subsequently performed. Cellulase activity determination was carried out to investigate whether the introduction of exogenous cellulase expression cassette will alter or enhance the cellulase production in PCR-positive transformants (Table S2). Among 22 positive transformants, the *celA* transformants A2-E and A2-2, the *celB* transformant B11-C2 and the *celC* transformants D1-B1 and D1-2(3) exhibited apparently and significantly (*p*<0.05) higher cellulase activities than the wild-type A-4 (Table 2). The FPAase activity of the above-mentioned five transformants increased by 22.69%–59.42% compared with that of wild-type strain. CMCase activity of the above-mentioned recombinant transformants was 2.26–8.49 times higher than that of wild-type A-4. Consistently, the fermented media of A2-E, A2-2, B11-C2, D1-B1 and D1-2(3) exhibited higher extracellular protein concentrations than that of wild-type A-4 and the control transformant harboring blank vector pPTR I.

**Table 2:** Cellulase activities, lipid production and cell growth of A2-E, A2-2, B11-C2, D1-B1 and D1-2(3).

Strains					Lipid production			
		Cellulase activity			SmF*		SSF**	
		FPAase(IU ml^−1^)	CMCase(IU ml^−1^)	Protein(µg ml^−1^)	Lipidyield (g l^−1^)	Biomass(mg l^−1^)	Lipid yield(g l^−1^)	LDM(%)***
Control+		5.01 (0.27)	19.71 (0.90)	89.64 (6.36)	-	-	-	-
WT++		5.20 (0.43)	32.94 (0.65)	102.17 (4.43)	0.63^b^ (0.04)	15.01 (0.27)	36.14^D^ (3.98)	26.05 (2.33)
*celA*	A2-E	7.32 (0.57)	134.69 (3.06)	141.45 (3.56)	0.69^b^ (0.07)	23.33 (3.33)	35.14^D^ (0.83)	32.05 (0.63)
	A2-2	8.02 (0.27)	312.56 (13.23)	181.93 (6.39)	1.47^a^ (0.05)	23.24 (3.02)	53.20^B^ (0.29)	31.20 (0.71)
*celB*	B11-C2	6.38 (0.17)	237.79 (16.18)	141.69 (7.73)	0.74^b^ (0.04)	17.50 (1.70)	45.62^C^ (5.95)	33.50 (2.68)
*celC*	D1-B1	8.29 (0.34)	107.33 (13.44)	146.27 (7.94)	1.27^a^ (0.24)	23.16 (3.60)	63.30^A^ (4.63)	25.85 (1.34)
	D1-2(3)	7.55 (0.51)	137.87 (14.69)	121.21 (0.17)	0.74^b^ (0.15)	13.73 (0.82)	41.26^C^ (1.31)	27.00 (2.12)

Incubation for cellulase activity measurement was conducted under submerged (SmF) conditions using wheat straw as substrate for 4 days. Lipid production and cell growth of transformants were determined under both submerged and solid-state conditions.

+ The control transformant introduced with the negative vector pPTRI without cellulase expression cassette.

++ The wild-type *A. oryzae* A-4.

*Submerged fermentation from wheat straw after 4 days.

**Solid state fermentation from wheat straw and bran mixture after 4 days.

***Loss in dry matter (LDM).

One-way ANOVA is used to test for differences: a, b means *p*<0.05; A, B, C, D means *p*<0.05; Values in brackets are standard errors.

Differences in cellulase secretion among 22 PCR-positive transformants harboring different cellulase genes were also concluded based on the results of Table S2. Both *celA* gene and *celB* gene encode endoglucanases, a kind of cellulase tending to hydrolyze amorphous cellulose such as carboxymethyl cellulose effectively [24]. The over-expression of endoglucanase offered the *celA* transformants and the *celB* transformants significantly (*p*<0.05) higher CMCase activities than other detected strains. It could be noted that insignificant (*p*>0.05) difference in CMCase activity was found between the *celA* transformants and the *celB* transformants. However, only the introduction of *celC* expression cassette into A-4 strain gave a promising positive effect on cellulase production though both *celD* gene and *celC* gene encode cellubiohydralase. Post Hoc comparisons using the Fisher LSD test further demonstrated that transformants integrated with *celA* gene and with *celC* gene exhibited significantly (*p*<0.05) higher average FPAase activity than other strains including *celB* transformants, *celD* transformants, the reference strain A-4 and the negative control transformant (transformed with blank vector pPTR I). Since the total cellulase activity is represented by FPAase activity in most cases, it could be speculated that it is more possible to get recombinant strains with improved cellulase secretion by introduction of *celA* gene and *celC* gene rather than *celB* gene and *celD* gene into *A. oryzae* A-4.

### Cellulosic lipids production of transformants with increased cellulase activity

The cellulosic lipids accumulating abilities of transformants A2-E, A2-2, B11-C2, D1-B1 and D1-2 (3) were assessed by using SmF media with wheat straw as the main substrate and SSF media with wheat straw and bran mixtures as the main substrate. Fermentations were performed for 4 days and the lipid production results were shown in Table 2. Under the SmF condition, the lipid yields of A2-2 and D1-B1 were 1.47 g l^−1^ and 1.27 g l^−1^, both of which were significantly (*p*<0.05) higher than those of other transformants and the wild-type A-4. Likewise, almost all transformants exhibited a significantly (*p*<0.05) higher lipid yield than the wild-type A-4 under the SSF condition, except A2-E. Among these transformants, D1-B1 exhibited a most robust lipid production and released a lipid yield of 63.30 mg gds^−1^ after 4 days’ fermentation under the SSF condition. In our previous report, wild-type *A. oryzae* A-4 gave a maximum lipid yield of 62.87 mg gds^−1^ after 6 days’ fermentation under an optimized SSF condition. The fermentation period of D1-B1 was shortened compared with that of the wild-type A-4. Besides, the lipid production of D1-B1 here was not under the optimized SSF condition, so it is potentially possible to be further improved after biochemical optimization. In conclusion, A2-2 and D1-B1 produced 1.33 times and 1.02 times higher amounts of lipids than wild-type A-4 after SmF of cellulose, while the lipid yields of A2-2 and D1-B1 increased by 35.22% and 59.57% compared to wild-type strains after SSF of wheat straw and bran mixtures. A2-2 and D1-B1 have an enhanced cellulosic lipid production and can be selected for subsequent characterization.

### Variability in metabolism associated to the introduction of cellulase gene

#### SmF using monosaccharides based carbon sources

**Figure 3:**
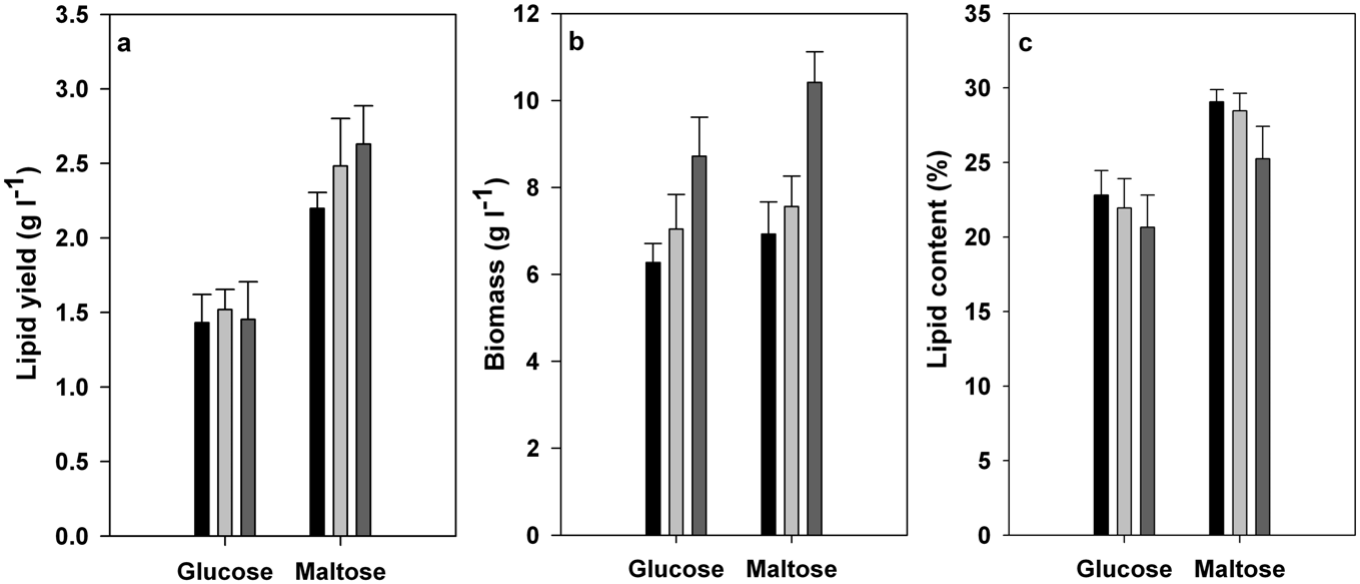
The lipid production (A, C) and cell growth (B) of A2-2 (black column), D1-B1 (gray column) and wild-type A-4 (dark gray column) in SmF experiments using glucose and maltose based media, respectively.

For traditional lipid production from either glucose or maltose, the cellulase over-expression exhibited little positive effects on the increase in lipid yields; the lipid production of A2-2 and D1-B1 on maltose media were ever poorer than that of wild-type A-4 (Fig. 3a). As shown in Fig. 3b, the overall biomass yields for both A2-2 and D1-B1 were lower than that for wild-type A-4 when cultured on glucose media and maltose media, which suggested that the introduction and expression of cellulase gene did inhibit the cell growth of the transformants cultured on *monosaccharide* based media. The metabolic burdens imposed by the cellulase over-expression on host strains could be expected to be a main cause for the reduced cell growth as cellulase expression is unnecessary for the cell growth of strain A-4 using glucose or maltose as carbon source. The simlar metabolic burdens imposed on host strains by the over-expression of foreign proteins have been previously reported by other researchers [25], [26].

The lipid content (%) presented the ratio of lipid yield in Fig. 3a and the corresponding biomass in Fig. 3b. Both A2-2 and D1-B1 had higher lipid contents than wild-type A-4 (Fig. 3c), which indicated that their lipid yields per unit of biomass have been improved. Therefore, it can be concluded that A2-2 and D1-B1 had a more robust lipid accumulation than wild-type A-4 when using glucose media and maltose media. The increased lipid accumulation in A2-2 and D1-B1 suggested that the insertion and over-expression of foreign cellulase gene affected the flux of carbon sources, and more carbon sources would be diverted into lipid biosynthesis.

#### SmF using cellulose based carbon sources

**Figure 4:**
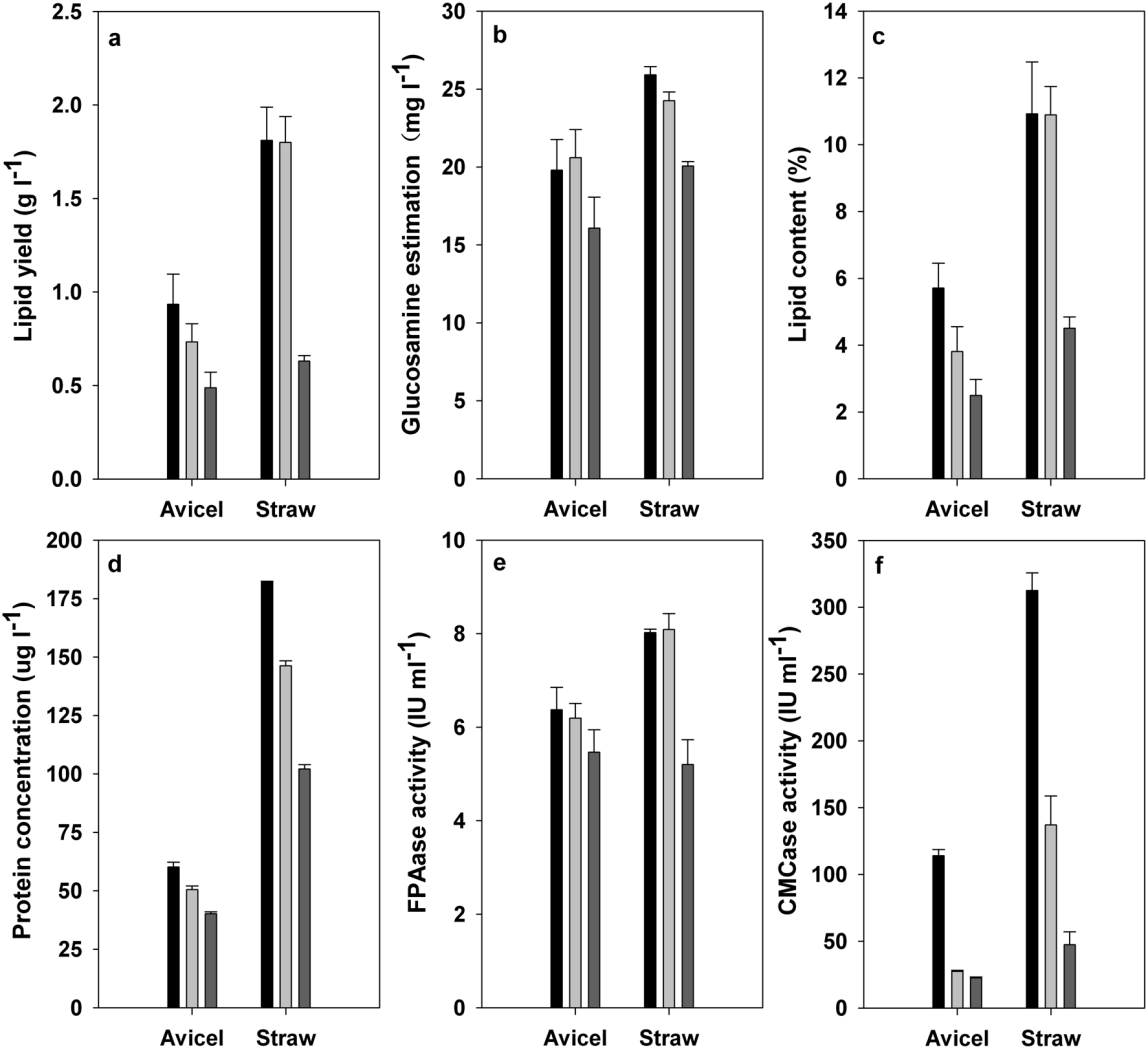
The lipid production (A, C), cell growth (B), cellulase secretion (E, F) and extracellular protein secretion (D) of A2-2 (black column), D1-B1 (gray column) and wild-type A-4 (dark gray column) in SmF experiments using avicel and straw based media, respectively.

The fermentation using cellulase based substrates did exhibit a different profile from that using monosaccharides based carbon sources. As shown in Fig. 4, both A2-2 and D1-B1 yielded a significantly higher amount of lipids than wild-type A-4. The introduction and expression of cellulase gene in strain A-4 enhanced not only lipid accumulation (Fig. 4c) but also the cell growth (Fig. 4b). The extracellular protein concentration, FPAase activity and CMCase activity from fermentation trails of A2-2 and D1-B1 by using both avicel and wheat straw were found to be significantly (*p*<0.05) and apparently higher than those from the trial of wild-type A-4 (Fig. 4d, 4e and 4f). The A2-2 with endoglucanase overexpressed exhibited higher CMCase activity than D1-B1 with cellubiohydralase overexpressed (Fig. 4f). However, insignificant (*p*<0.05) differences in the FPAase activities were found between D1-B1 and A2-2 (Fig. 4c). It is well-known that sufficient available monosaccharide is indispensible for robust cell growth. Cellulase is necessary for cellulose decomposition in CBP from lignocellulose. Thus, it could be suggested that the enhanced cell growth and lipid production (Fig. 4a) in A2-2 and D1-B1 were attributed to their improved cellulase secretion owing to the over-expression of foreign cellulase. Moreover, we noted that positive effects of the cellulase gene integration on fermentation were more apparent in wheat straw trails than that in avicel trails. It appeared that the improving effects of cellulase over-expression on the lipid production were more effective when using wheat straw as substrates, although more researches should be performed for this conclusion. Overall, our work indicates the promising utilization of A2-2 and D1-B1 for lipid production from wheat straw.

#### SSF using wheat straw and bran mixtures

**Figure 5:**
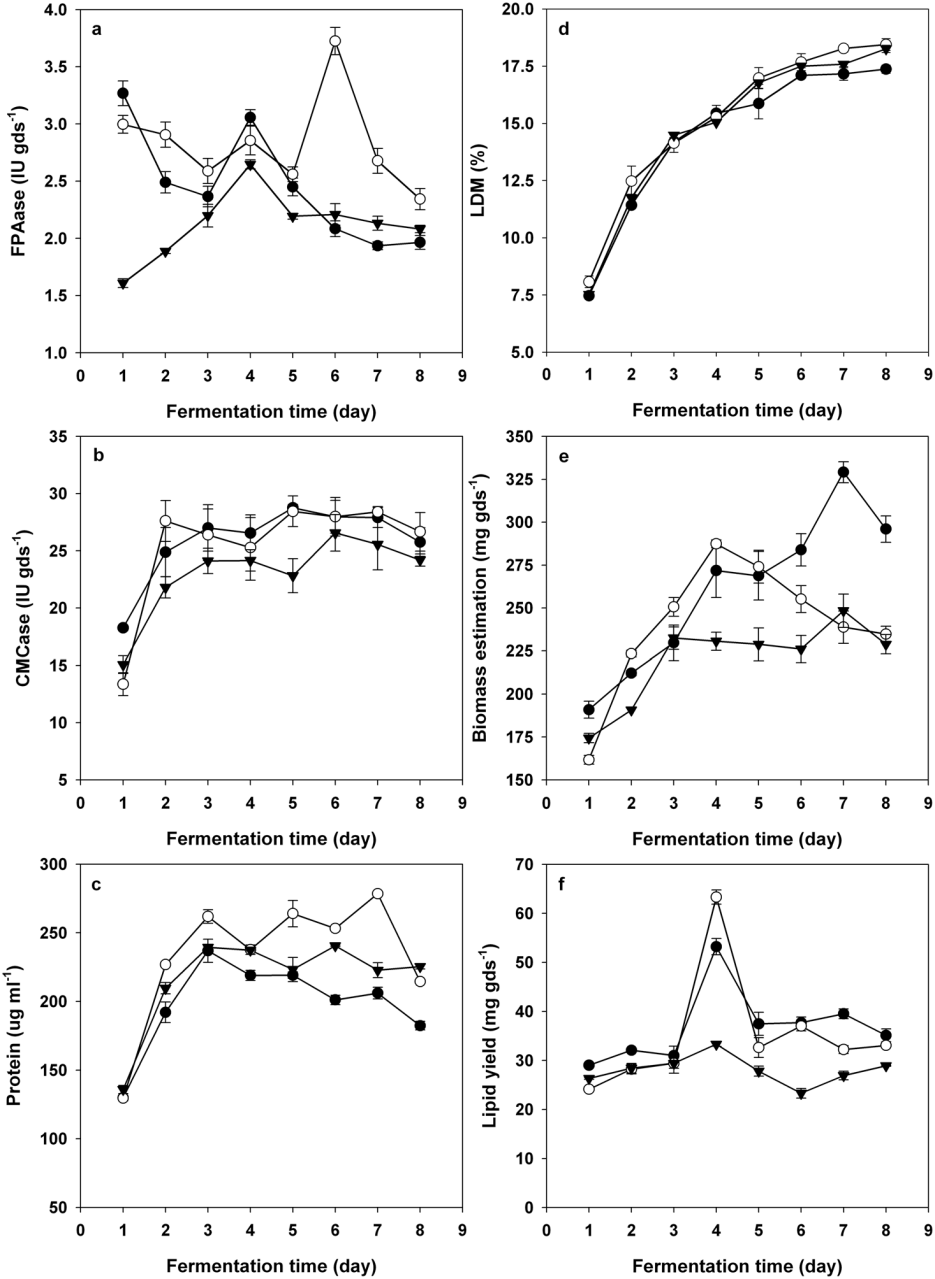
Solid-state fermentation of wheat straw and bran mixture by D1-B1 (blank circle), A2-2 (closed circle) and wild-type A-4 (closed triangle). (A) Time courses of the filter paper activity (FPAase activity); (B) Time courses of the CMCase activity; (C) Time courses of the extracellular protein concentration; (D) Time courses of the loss in dry matter (LDM); (E) Time courses of the cell growth; (F) Time courses of the lipid yield.

As shown in Fig. 5, the ferment strains exhibited cellulase secretion and lipid production both in the order of D1-B1>A2-2>wild-type A-4. SSF profiles using wheat straw and bran mixtures as substrates were characterized in detail for A2-2, D1-B1 and wild-type A-4 as follows.

In terms of FPAase activity (Fig. 5a), introduction of cellulase gene into cells changed the cellulase secretion in microbes. In the early stage of fermentation (within 4 days), both A2-2 and D1-B1 showed an extremely high cellulase activity at the first 24 hours, which then decreased gradually. Different from that observed in A2-2 and D1-B1, the cellulase activity of the wild-type A-4 increased slowly and gradually throughout the early fermentation stage. It seemed that the introduced cellulase genes in both A2-2 and D1-B1 gave a robust expression in the early fermentation stage; this may be the reason why A2-2 harboring endoglucanase gene showed a higher CMCase activity than either D1-B1 harboring cellubiohydralase gene or wild-type A-4 at the first 24 hours (Fig. 5b). The strong cellulase over-expression that tends to occur in the early fermentation stage such as within 24 hours could be attributed to the characteristics of the *hlyA* promoter in most cases. The similar phenomenon had been reported by Bando et al. [14], who firstly showed that the endoglucanase gene expressed under the control of the *hlyA* promoter would give a continued increase of endoglucanase activity within the 48 hours but a gradual decrease after 48 hours. Besides, the introduction of *celC* gene attributed to a particular phenomenon in FPAase activity of D1-B1 that FPAase activity of D1-B1 showed a sharp increase during the later fermentation stage from the 5^th^ day to the 7^th^ day. Thus, the detail metabolic profiles between A2-2 harboring *CelA* gene and D1-B1 harboring *CelC* gene has become different though the cellulase overexpression led to increased cellulase secretion and lipid production in both A2-2 and D1-B1.

The extracellular protein concentrations were in the order of D1-B1>WT>A2-2 (Fig. 5c). According to those found in fermentations by using monosaccharide based carbon source, it is supposed that the lower extracellular protein concentration in A2-2 could attribute to its reduced endogenous protein secretion due to the metabolic burden imposed by the over-expression of foreign cellulase genes.

There is no apparent difference in LDM among the three detected strains (Fig. 5d). However, A2-2 and D1-B1 have higher growth rates than the reference strain A-4. The biomass of wild-type strains increased slowly with the proceeding of the time and decreased after the 7^th^ day, while the biomass of D1-B1 increased rapidly and reached the maximum on the 4^th^ day. Different from that of both D1-B1 and the reference strain, the cell growth of A2-2 increased rapidly and stably with the increase of fermentation time. Although the growth rate of A2-2 was lower than D1-B1, the maximum biomass of A2-2 was found to be higher than that of D1-B1 (Fig. 5e). A2-2, D1-B1 and the reference strain showed similar changing trends of lipid yields, and the lipid yields of all strains increased during the early fermentation and reached the maximum on the 4^th^ day. Furthermore, we noted that that the fermentation period from 72 h to 96 h might be important for the lipid accumulation (Fig. 5f). The higher increasing rates of lipid yield from 72 h to 96 h related closely with the final lipid yields in A2-2 and D1-B1.

Generally, the transformation and over-expression of foreign cellulase gene attributed to the enhancement of lipid accumulation in A2-2 and D1-B1. Although over-expression of foreign cellulase gene in strain A-4 imposed metabolic burden on cells, it offers A2-2 and D1-B1 higher biomass and lipid yields during cellulosic lipids production. Polymers decomposition mainly depends on the hydrolytic activity of the microbes [13], so it is understandable that more sufficient available carbon sources provided for microbes will lead to higher theoretical yields of lipids. As the conventional substrate for SCOs production was glucose, the current reports regarding genetic manipulations for enhanced SCOs production mainly centered on the modification of lipid biosynthesis pathway such as regulating the TAG biosynthesis pathway enzymes and blocking competing pathways (e.g., β-oxidation) [27]. According to the previous reports and the current investigation, we proposed that combined manipulation of the hydrolytic ability and lipid biosynthesis pathway in microbes would be more effective to promote cellulosic lipid production in CBP system.

### Preliminarily study of the cellulase gene integration profiles in transformants

**Figure 6:**
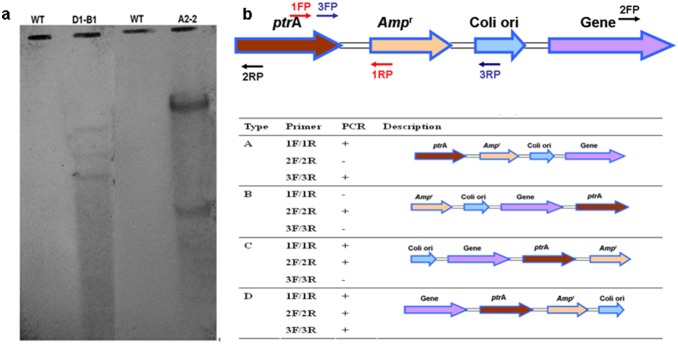
Preliminarily study of cellulase gene integration profiles in A2-2 and D1-B1, respectively. (A) Southern blot analysis. A part of *Amp* ^r^ gene (0.7-kb) was amplified by PCR and used as the probe. (B) Primers designed for the insertion analysis of the target gene in A2-2 (*celA*) and D1-B1 (*celC*), and the theoretical results of PCR using the designed primers.

Although both A2-2 and D1-B1 showed enhanced lipid production, the detail metabolic profiles of A2-2 harboring *celA* gene and D1-B1 harboring *celC* gene were found to be different. Integration profiles of cellulase gene in the two transformants were investigated preliminarily in this work. The pPTRI based vector is integrated with the host genomic DNA by non-homologous recombination. For non-homologous integration of transforming vectors in the fungus, the complete vector backbone would often be integrated into the genomic DNA. Thus, Ampicillin resistance gene (*Amp* ^r^) was used instead of cellulase gene as the probe for southern blot analysis. For southern blot analysis, genomic DNA extracted from A2-2 and D1-B1 was digested with *Hind*III and subsequent hybridization with *Amp* ^r^ probe. The hybridization results would provide an estimate of transgene copy number and the number of insertion loci. According to the results shown in Fig. 6a, it can be estimated that A2-2 contains two copies of the transgene while D1-B1 has three copies (Fig. 6a). Subsequently, three primer sets were designed to investigate the integration type of “cellulase expression cassette” in transformants (Fig. 6b). According to PCR results using genomic DNA of A2-2, D1-B1 and wild-type strain A-4 as templates (Fig. S2), the insertion type of “cellulase expression cassette” in A2-2 was suggested to be type A. However, one strain can receive several plasmids and every plasmid could integrate with the host genome in different types. Thus, the insertion type in D1-B1 is difficult to determine using PCR, and the integration type of “cellulase expression cassette” in D1-B1 could be type D or the combination of several types. Overall, identification of the insertion type of exogenous expression cassette by using one method such as PCR could be insufficient for some transformants (e.g. D1-B1). Although more researches and technologies are needed to answer this question, the obtained information of the insertion type of “cellulase expression cassette” in both A2-2 and D1-B1 is important for the elucidation of the insertion loci of the transgene, which could be guidance for primer design in Genome Walking. Besides, it has been reported that different insertion of genes on the chromosome has significant effects on expression levels of foreign enzymes, which is mediated through the interferences from local sequences, gene orientation, and insertion position relative to the chromosomal origin of replication [28]. Thus, it could be proposed that the different metabolic profiles between A2-2 and D1-B1 during cellulosic lipid production could attributed to not only their different transgene but also biological impact of different integration profiles in transformants.

## Conclusion

This work indicated that the enhancement of lipid production can be promisingly feasible by regulating cellulase secretion of natural CBP producers, although lipid production was not affected and controlled by cellulase activity alone. For strain A-4, it is more possible to get strains with increased cellulase activity by the introduction of *celA* and *celC* gene than *celB* and *celD* gene. *celA* transformant A2-2 and *celC* transformant D1-B1 were demonstrated to be good candidates for efficient conversion of biomass into lipids, exhibiting significantly higher lipid yields than the reference strain using cellulosic substrates. The foreign cellulase introduction contributed to not only enhanced simultaneous saccharification but also improved lipid accumulation in A2-2 and D1-B1.

## Supporting Information

Figure S1 PCR results using the genomic DNA of transformants and wild-type A-4.
(DOC)

Figure S2 PCR detection of the genomic DNA of the A2-2, D1-B1 and wild-type A-4 by using the primers as described in Table S1.
(DOC)

Table S1 Primers used in this study.
(DOC)

Table S2 Cellulase activities of 22 PCR-positive transformants and the reference strains.
(DOC)
