# Supplementary material for: Engineering Aspergillus oryzae A-4 through the Chromosomal Insertion of Foreign Cellulase Expression Cassette to Improve Conversion of Cellulosic Biomass into Lipids
Source: PLoS One. 2014 Sep 24;9(9):e108442. doi: 10.1371/journal.pone.0108442 (PMC4177402; doi:10.1371/journal.pone.0108442)
Supplement: Figure S1 — PCR results using the genomic DNA of transformants and wild-type A-4. (DOC) [file pone.0108442.s001.doc]

**
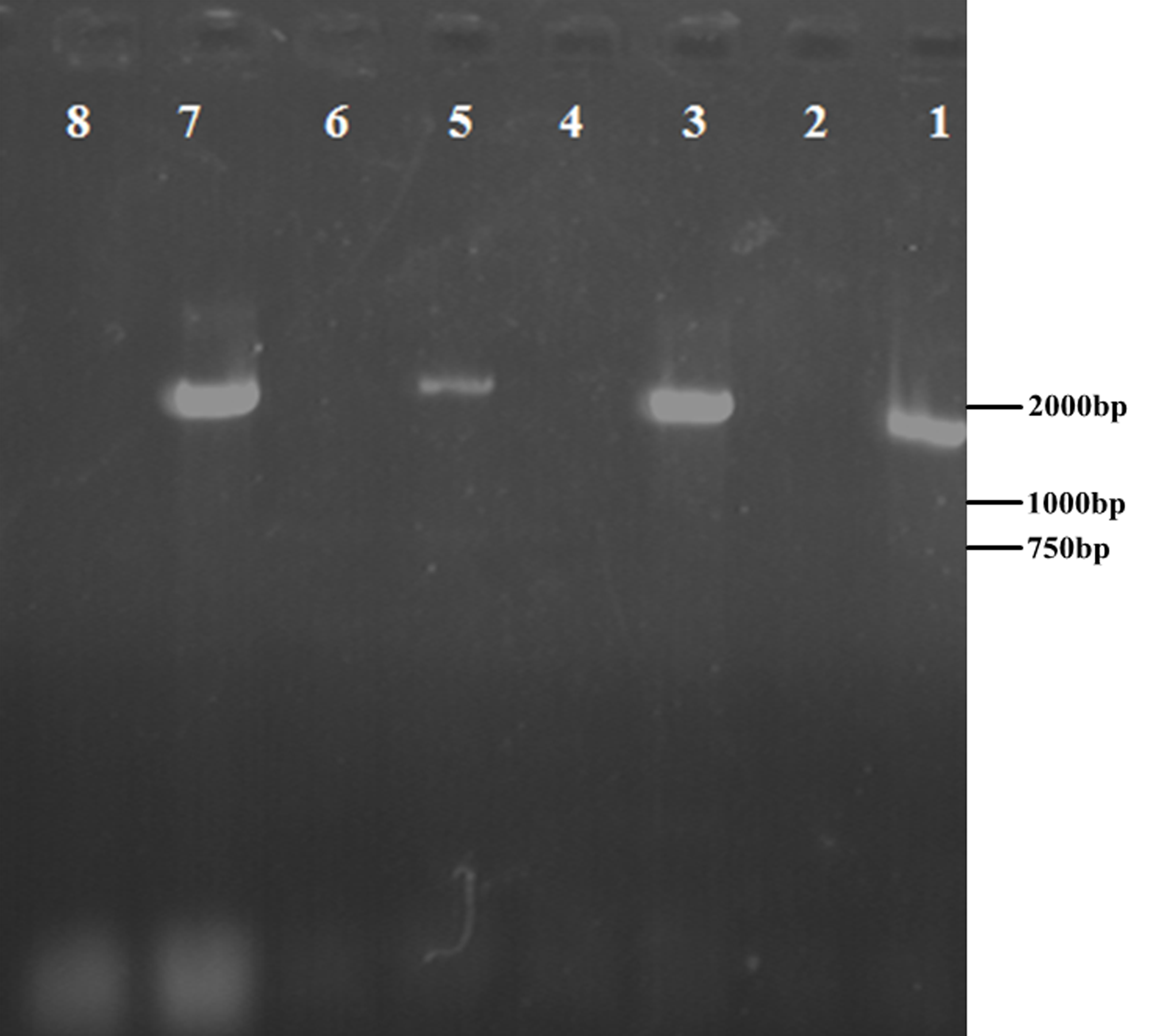
**

**Figure S1** PCR results using the genomic DNA of transformants and wild-type A-4. 1: *celA* transformants using the primer set of FP1-hlyA-Rec/ RP2-CelA-Rec; 2: wild-type A-4 (WT) using the primer set of FP1-hlyA-Rec/ RP2-CelA-Rec; 3: *celB* transformants using the primer set of FP1-hlyA-Rec/ RP2-CelB-Rec; 4: WT using the primer set of FP1-hlyA-Rec/ RP2-CelB-Rec; 5: *celD* transformants using the primer set of FP1-hlyA-Rec/ RP2-CelD-Rec; 6: WT using the primer set of FP1-hlyA-Rec/ RP2-CelD-Rec; 7: *celC* transformants using the primer set of FP1-hlyA-Rec/ RP2-CelC-Rec; 8: WT using the primer set of FP1-hlyA-Rec/ RP2-CelC-Rec.
