# Supplementary material for: Engineering Aspergillus oryzae A-4 through the Chromosomal Insertion of Foreign Cellulase Expression Cassette to Improve Conversion of Cellulosic Biomass into Lipids
Source: PLoS One. 2014 Sep 24;9(9):e108442. doi: 10.1371/journal.pone.0108442 (PMC4177402; doi:10.1371/journal.pone.0108442)
Supplement: Figure S2 — PCR detection of the genomic DNA of the A2-2, D1-B1 and wild-type A-4 by using the primers as described in Table S1. (DOC) [file pone.0108442.s002.doc]

**
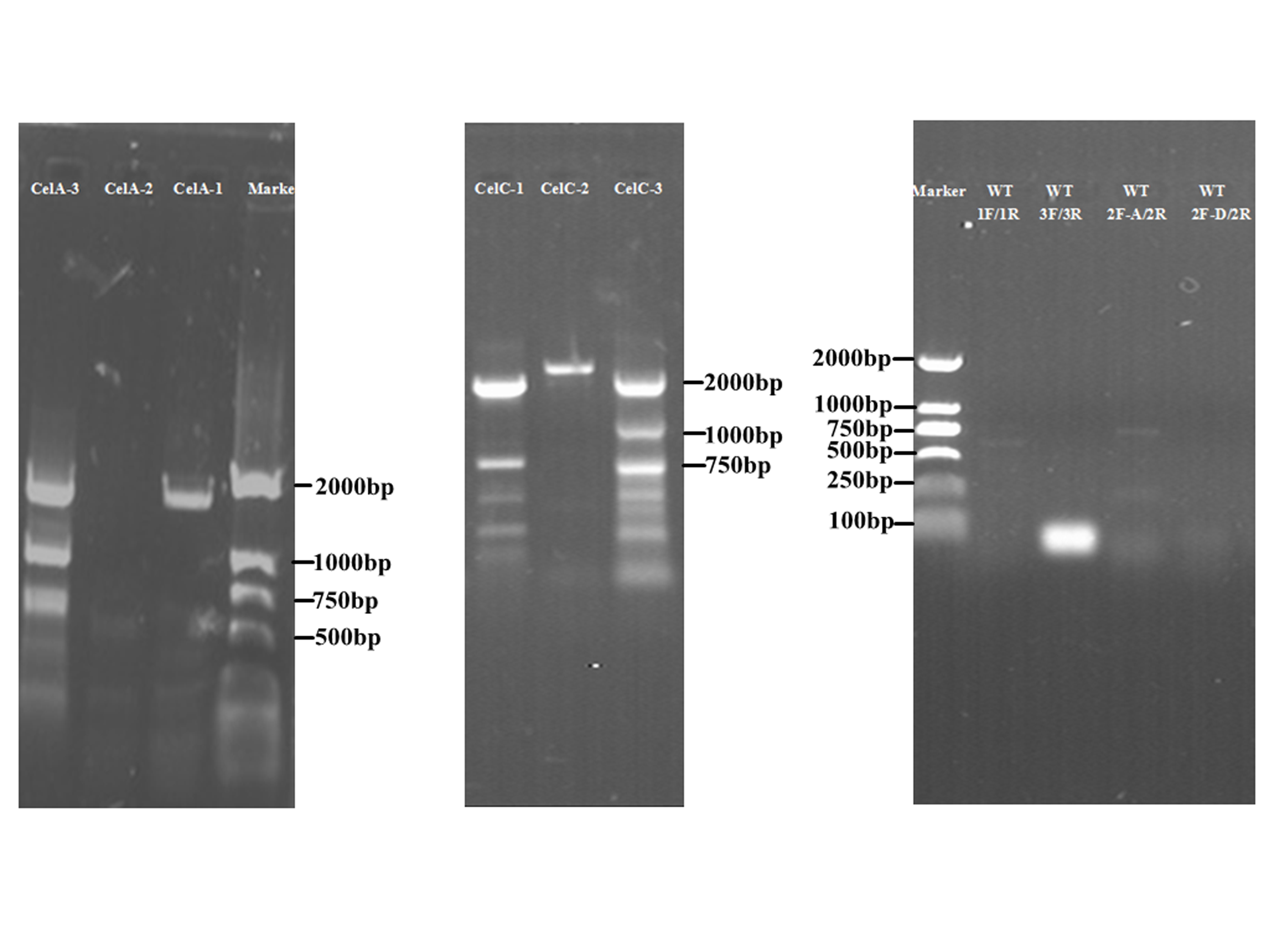
**

**Figure S2** PCR detection of the genomic DNA of the A2-2, D1-B1 and wild-type A-4 by using the primers as described in Table S1. PCR results for A2-2 (Lanes CelA-1, CelA-2 and CelA-3) and D1-B1 (Lanes CelC-1, CelC-2 and CelC-3) were shown. The bands of the expect size were cut out of the gel, extracted and sequenced. The sequence alignment results confirmed that the DNA sequences of the PCR fragments were consistent with our target DNA. No products (Lane WT) of the expect size were amplified from the genomic DNA of wild-type A-4 by using the primers as described in Table S1. The primer set of 1F/1R was used for Lanes CelA-1, CelC-1 and WT 1F/1R. The primer set of 2F-A/2R was used for Lanes CelA-2 and WT 2F-A/2R; The primer set of 2F-D/2R was used for Lanes CelC-2 and WT 2F-D/2R; The primer set of 3F/3R was used for Lanes CelA-3, CelC-3 and WT 3F/3R.
