# Supplementary material for: Engineering Aspergillus oryzae A-4 through the Chromosomal Insertion of Foreign Cellulase Expression Cassette to Improve Conversion of Cellulosic Biomass into Lipids
Source: PLoS One. 2014 Sep 24;9(9):e108442. doi: 10.1371/journal.pone.0108442 (PMC4177402; doi:10.1371/journal.pone.0108442)
Supplement: Table S1 — Primers used in this study. (DOC) [file pone.0108442.s003.doc]

**Table S1** Primers used in this study

| Name | Sequence (5′-3′) |
| --- | --- |
| For construction of plasmid with the *amyA* terminator | |
| FP-TamyA-XK | GGGGTACCCTCGAGGATCTGTAGTAGCTCGTGAAGG (*Kpn*I and *Xho*I) |
| RP-TamyA-K | GGGGTACCTTTCCTATAATAGACTAGCGTGCTT (*Kpn*I) |
| For construction of target gene expression vector | |
| FP1-hlyA-Rec | gaccatgattacgccAGATCTTACAGCATGGTCTGGATTCCAA (*Bgl*II) |
| RP1-hCelA-overlap | caatgagagcttcatTTGTGGTGTGAAGGGTGATTG |
| FP2-CelA-overlap | acccttcacaccacaaATGAAGCTCTCATTGGCACTT |
| RP2-CelA- Rec | tcctctagagtcgacAGATCTTTAGTTGACACTGGCAGTCC (*Bgl*II) |
| RP1-hCelB-overlap | gagtgtccagatcatTTGTGGTGTGAAGGGTGATTG |
| FP2-CelB-overlap | cccttcacaccacaaATGATCTGGACACTCGCTCC |
| RP2-CelB-Rec | tcctctagagtcgacAGATCTCTAATGCCTGTAGGTAGATCC (*Bgl*II) |
| RP1-hCelD-overlap | caccccggtgttcatTTGTGGTGTGAAGGGTGATTG |
| FP2-CelD-overlap | cccttcacaccacaaATGAACACCGGGGTGTTTCTCTC |
| RP2-CelD-Rec | tcctctagagtcgacAGATCTTTAAGCTGGAGCGTCGAAAG (*Bgl*II) |
| FP1-hCelC -Rec | gaccatgattacgccCTCGAGTACAGCATGGTCTGGATTCCAA (*Xho*I) |
| RP1-hCelC-overlap | ggaaagggaagccatTTGTGGTGTGAAGGGTGATTG |
| FP2-CelC-overlap | cccttcacaccacaaATGGCTTCCCTTTCCCTCT |
| RP2-CelC-Rec | tcctctagagtcgacCTCGAGTCAGCTCTTGAAGGTGGAGC (XhoI) |
| RP1-hGFP-overlap | gcccttgctcaccatTTGTGGTGTGAAGGGTGATTG |
| FP2-GFP-overlap | cccttcacaccacaaATGGTGAGCAAGGGCG |
| RP2-GFP-Rec | tcctctagagtcgacAGATCTTTACTTGTACAGCTCGTCCATG (*Bgl*II) |
| Template DNA preparation for DIG-High Prime labeling | |
| South-Amp-FP4 | ATTCAACATTTCCGTGTCGC |
| South-Amp-RP4 | CTTTTGGCTGGACTCTCACAAT |
| PCR primers for integration profile analysis | |
| 1F | TTGAACTCGTCCCACTTGCCAC |
| 1R | CCGGCTCCAGATTTATCAGCAA |
| 3F | AAGTATTCCATGACTTCCATCG |
| 3R | GAGCGAGGTATGTAGGCGGTGC |
| 2F-A2-2 | GACGTGGAGTGGAGTCAGGATA |
| 2F-D1-B1 | CCTGCGGTGGCACATATAG |
| 2R-D-A | GAGGTTACTAAGGGCTTGATAA |
