# Supplementary material for: Engineering Aspergillus oryzae A-4 through the Chromosomal Insertion of Foreign Cellulase Expression Cassette to Improve Conversion of Cellulosic Biomass into Lipids
Source: PLoS One. 2014 Sep 24;9(9):e108442. doi: 10.1371/journal.pone.0108442 (PMC4177402; doi:10.1371/journal.pone.0108442)
Supplement: Table S2 — Cellulase activities of 22 PCR-positive transformants and the reference strains. (DOC) [file pone.0108442.s004.doc]

**Table S2** Cellulase activities of 22 PCR-positive transformants and the reference strains. Incubation was conducted under SmF conditions using wheat straw as the substrate for 4 days.

| Transformants | | FPAase (IU ml^-1^) | CMCase (IU ml^-1^) | Protein (μg ml^-1^) |
| --- | --- | --- | --- | --- |
| WT ^a^ | | 5.20±0.43 | 32.94±0.65 | 102.17±4.43 |
| Control ^b^ | | 5.01±0.27 | 19.71±0.90 | 89.64±6.36 |
| *celA* | A2-A | 6.16±0.46 | 56.20±3.33 | 112.05±9.71 |
|  | A2-1(1) | 5.34±0.29 | 112.56±8.27 | 115.43±5.16 |
|  | A2-1(2) | 5.70±0.06 | 108.36±2.06 | 100.25±0.68 |
|  | A2-E | 7.32±0.57 | 134.69±3.06 | 141.45±3.56 |
|  | A2-2 | 8.02±0.27 | 312.56±13.23 | 181.93±6.39 |
| *celB* | B11-1(2) | 4.37±0.08 | 87.54±4.51 | 98.80±4.26 |
|  | B11-4 | 4.90±0.28 | 105.02±5.41 | 92.78±2.26 |
|  | B11-2(2) | 4.78±0.25 | 108.56±9.59 | 112.29±8.93 |
|  | B11-E2(1) | 5.08±0.21 | 118.42±6.25 | 109.88±6.82 |
|  | B11-C2 | 6.38±0.17 | 237.79±16.18 | 141.69±7.73 |
| *celC* | D1-B1 | 8.29±0.34 | 107.33±13.44 | 146.27±7.94 |
|  | D1-1(1) | 5.63±0.08 | 25.92±0.32 | 90.849±2.90 |
|  | D1-2(2) | 5.94±0.18 | 31.52±1.32 | 114.70±10.22 |
|  | D1-A | 5.25±0.14 | 30.34±2.15 | 99.28±1.56 |
|  | D1-2(1) | 6.13±0.25 | 102.60±7.97 | 100.97±2.62 |
|  | D1-2(3) | 7.55±0.51 | 137.87±14.69 | 121.21±0.17 |
| *celD* | C4-1(2) | 4.87±0.17 | 42.11±1.74 | 126.75±5.04 |
|  | C4-3 | 5.06±0.14 | 31.35±4.72 | 99.04±2.04 |
|  | C4-C1 | 5.37±0.08 | 79.40±8.92 | 120.00±1.87 |
|  | C4-1(1) | 4.46±0.25 | 88.28±9.57 | 87.96±1.04 |
|  | C4-4 | 4.93±0.13 | 133.12±2.06 | 112.05±2.07 |
|  | C4-B | 5.40±0.15 | 147.03±7.96 | 135.19±3.57 |

^a^ The wild-type *A. oryzae* A-4

^b^ The control transformant introduced with the negative vector pPTRI without cellulase expression cassette
